# Monocyte subsets in bone marrow grafts may contribute to a low incidence of acute graft‐vs‐host disease for young donors

**DOI:** 10.1111/jcmm.15557

**Published:** 2020-06-30

**Authors:** Qi Wen, Hong‐Yan Zhao, Wei‐Li Yao, Yuan‐Yuan Zhang, Hai‐Xia Fu, Yu Wang, Lan‐Ping Xu, Xiao‐Hui Zhang, Yuan Kong, Xiao‐Jun Huang

**Affiliations:** ^1^ Department of Hematology, Nanfang Hospital Southern Medical University Guangzhou China; ^2^ Peking University People’s Hospital Peking University Institute of Hematology National Clinical Research Center for Hematologic Disease Beijing Key Laboratory of Hematopoietic Stem Cell Transplantation Collaborative Innovation Center of Hematology Peking University Beijing China

**Keywords:** acute graft‐vs‐host disease, allogeneic haematopoietic stem cell transplantation, donor graft, monocytes

## Abstract

Young donors are associated with a lower cumulative incidence of acute graft‐vs‐host disease (aGVHD) after allogenic haematopoietic stem cell transplantation (allo‐HSCT) than old donors. Although grafts are harvested from healthy donors, it is unclear whether donor age is associated with aGVHD occurrence owing to its effect on cell compositions in grafts. Moreover, the differences in monocyte subsets in grafts between young and old donors and the association between monocyte subsets in bone marrow (BM) grafts and aGVHD remain to be elucidated. In the current study, non‐classical monocytes and the CD4^+^/CD8^+^ T cell ratio were remarkably decreased in BM grafts in donors <30 years old. Multivariate analysis further revealed that the level of non‐classical monocytes in BM grafts (≥0.31 × 10^6^/kg) was an independent risk factor for the occurrence of II‐IV aGVHD. In summary, our data indicate that non‐classical monocytes in BM grafts may help identify patients at high risk for aGVHD after allo‐HSCT. Although further validation is required, our results suggest that the low level of non‐classical monocytes and a low ratio of CD4^+^/CD8^+^ T cell in BM grafts may be correlated with the lower incidence of aGVHD in young donors.

## 1 INTRODUCTION

Allogeneic haematopoietic stem cell transplantation (allo‐HSCT) provides a potential curative therapy for patients with haematological diseases. However, acute graft‐vs‐host disease (aGVHD) remains a major complication after allo‐HSCT.^1^, ^2^, ^3^ The consensus for donor selection suggests that young donors are a better choice for patients, as they are associated with a lower incidence of aGVHD after allo‐HSCT than old donors.^4^, ^5^, ^6^ Several studies in HLA‐matched transplants have shown a lower incidence of aGVHD using grafts from young donors.^7^, ^8^ The impact of donor age has been confirmed in the setting of haploidentical stem cell transplantation (haplo‐SCT).^6^ Wang et al reported a lower incidence of aGVHD associated with young donors (<30 years old) in haplo‐SCT based on immune tolerance induced by granulocyte colony‐stimulating factor (G‐CSF) and antithymocyte globulin (ATG).^9^ González‐Vicent et al demonstrated a lower incidence of aGVHD after T cell‐depleted haplo‐SCT when using grafts from younger donors (<40 years old).^10^ Nevertheless, the underlying reason why young donors are associated with a lower incidence of aGVHD is still unknown.

The pathogenesis of aGVHD is commonly believed to be caused by exaggerated and undesirable immune responses in which there is a complex interplay between the donor cells and recipient cells. It has been reported that the different cell compositions in donor grafts are involved in the pathogenesis of aGVHD.^11^, ^12^, ^13^, ^14^ The increased ratio of CD4^+^/CD8^+^ T cells in donor bone marrow (BM) grafts is often utilized as a biomarker for a high incidence of aGVHD. Moreover, our recent study reported that an imbalance in macrophage polarization in donor BM grafts, characterized by a high M1/M2 macrophage ratio, exhibited a high incidence of aGVHD.^15^ These studies suggest that the cell compositions in donor grafts may help to identify patients who are at high risk for aGVHD.

Given that grafts are harvested from healthy donors, donor age has been reported to be associated with the cell compositions in donor grafts. Yakoub‐Agha et al reported that CD8^+^‐naïve T cells in grafts are negatively associated with donor age, whereas the ratio of CD4^+^/CD8^+^ T cells and CD8^+^ effector memory T cells in grafts are positively associated with donor age.^16^ Furthermore, a high percentage of CD14^+^ monocytes was reported in grafts of young donors.^17^ In humans, circulating monocytes are classified into three subsets: classical, intermediate and non‐classical monocytes.^18^, ^19^ Classical monocytes are highly phagocytic and are important scavenger cells. Intermediate monocytes have antigen presentation and angiogenesis functions. Non‐classical monocytes demonstrate proinflammatory behaviour and secrete inflammatory cytokines in response to infection. In this regard, the imbalance in monocyte subsets has been reported to play a critical role in the occurrence and development of many inflammatory disorders. These findings suggest that the imbalance in monocyte subsets is a promising predictor for risk stratification in inflammatory diseases.^20^, ^21^, ^22^, ^23^, ^24^, ^25^, ^26^ However, the differences in monocyte subsets between young and old donors and the association between monocyte subsets in BM grafts and aGVHD remain to be elucidated.

Therefore, the current study was performed to determine whether donor age is associated with aGVHD occurrence owing to its effect on cell compositions in BM grafts. Our aim was to provide new insights into why young donors are a better choice for patients undergoing allo‐HSCT than old donors.

## 2 MATERIALS AND METHODS

### 2.1 Patients and their healthy donors

A total of 83 patients who underwent allo‐HSCT and their own healthy donors were enrolled at Peking University People's Hospital. The donor cohort comprised 59 males and 24 females, aged 16‐63 years old (median, 39 years old). As shown in Table 1, the enrolled donors were designated into young (age < 30 years), middle‐aged (30 years ≤ age≤45 years) and old (age > 45 years) donor groups. Blood cell counts including white blood cell (WBC), neutrophils, lymphocytes and monocytes in peripheral blood (PB) of healthy donors are analysed at three‐time points: before G‐CSF mobilization, before G‐CSF‐mobilized BM (G‐BM, on the fourth day after G‐CSF mobilization) harvesting and before G‐CSF‐mobilized peripheral blood (G‐PB, on the fifth day after G‐CSF mobilization) apheresis. Most of the characteristics including the underlying diseases of their related patients showed no significant differences among the three donor age groups, whereas the lymphocyte counts were significantly lower in middle‐aged donor group (Table 1). Subsequently, the effect of the monocyte subsets in BM grafts on the occurrence of aGVHD was evaluated.

**Table 1:** Characteristics of donors and their related patients

Characteristics	Young donor (n = 24)	Middle‐aged donor (n = 35)	Old donor (n = 24)	*P* *	*P* **	*P* ***
Gender, male/female	15/9	25/10	19/5	.47	.20	.50
Weight (kg)^a^	67.5 (47‐95)	71 (47‐90)	69 (45‐100)	.33	.82	.33
BMI (kg/m^2^)^a^	22.81 (18.91‐30.49)	24.49 (19.83‐29.88)	23.17 (16.94‐33.41)	.27	.61	.66
Blood cell counts (before G‐CSF mobilization)						
WBC (×10^9^/L)^a^	6.54 (3.86‐12.66)	5.93 (3.89‐9.95)	5.86 (3.54‐9.28)	.12	.18	.96
Neutrophils (×10^9^/L)^a^	3.15 (1.97‐7.80)	3.28 (1.51‐7.67)	3.40 (1.64‐5.27)	.42	.34	.83
Lymphocytes (×10^9^/L)^a^	2.30 (1.22‐3.95)	1.89 (0.92‐3.60)	2.08 (1.39‐3.30)	.02	.09	.45
Monocytes (×10^9^/L)^a^	0.44 (0.17‐0.86)	0.45 (0.21‐0.60)	0.44 (0.17‐0.74)	.43	.84	.55
Blood cell counts (before G‐BM harvesting)						
WBC (×10^9^/L)^a^	33.05 (14.90‐47.50)	31.29 (15.60‐44.90)	31.03 (21.87‐45.00)	.26	.80	.38
Neutrophils (×10^9^/L)^a^	27.20 (12.60‐40.70)	26.63 (13.30‐38.90)	27.34 (18.00‐38.20)	.27	.89	.32
Lymphocytes (×10^9^/L)^a^	3.05 (1.38‐4.90)	2.70 (1.50‐5.29)	2.67 (0.87‐4.40)	.21	.24	.90
Monocytes (×10^9^/L)^a^	1.59 (0.90‐4.20)	3.44 (1.51‐7.80)	1.68 (0.81‐3.00)	.16	.95	.14
Blood cell counts (before G‐PB harvesting)						
WBC (×10^9^/L)^a^	42.00 (31.27‐60.30)	41.70 (22.03‐57.47)	38.00 (28.40‐58.30)	.41	.21	.66
Neutrophils (×10^9^/L)^a^	36.4 (27.15‐54.10)	37.10 (27.15‐54.10)	33.45 (24.5‐50.9)	.43	.21	.65
Lymphocytes(×10^9^/L)^a^	3.10 (2.20‐4.60)	3.00 (1.30‐5.50)	3.17 (1.66‐6.93)	.50	.56	.26
Monocytes (×10^9^/L)^a^	2.10 (1.00‐4.00)	2.10 (0.90‐4.02)	1.81 (1.00‐3.40)	.53	.14	.30
The underlying diseases of their related patients						
AML	13	25	17	.17	.23	.96
ALL	10	9	5	.20	.12	.67
MDS	1	1	2	.78	.55	.35

Abbreviations: G‐BM, G‐CSF‐primed bone marrow; G‐CSF, granulocyte colony‐stimulating factor; G‐PB, G‐CSF‐primed peripheral blood; WBC, white blood cell.

^a^ Data are reported as median (range).

* *P‐*value between young and middle‐aged donors.

** *P‐*value between young and old donors.

*** *P‐*value between middle‐aged and old donors.

The current study was approved by the Ethics Committee of Peking University People's Hospital, and written informed consent was obtained from all patients and donors in compliance with the Declaration of Helsinki.

### 2.2 Transplantation protocols

Donor selection, conditioning therapy, graft harvesting and the prevention of GVHD have been described previously.^27^, ^28^, ^29^ Donors were ranked based on the best HLA match, age (younger preferred) and donor‐recipient sex (same preferred). Donors were injected subcutaneously with G‐CSF at 5 μg/kg daily for five consecutive days. For haplo‐SCT, recipients were treated with a modified busulfan/cyclophosphamide plus ATG regimen before the infusion of unmanipulated G‐BM and G‐PB. GVHD prophylaxis was performed with cyclosporine, mycophenolate mofetil and short‐course methotrexate. All transplantation recipients received cyclosporine A (CsA), mycophenolate mofetil (MMF) and short‐term methotrexate (MTX) as GVHD prophylaxis. The dosage of CsA was 2.5 mg/kg/d IV from day 9 until bowel function returned to normal, at which point, patients were switched to oral CsA. Every 12 hours, 0.5 g of MMF was administered orally from day 9 and was discontinued after engraftment. MTX was administered intravenously at 15 mg/m^2^ on day 1 and then at 10 mg/m^2^ on days 3, 5 and 11 in haplo‐SCT.

### 2.3 Clinical definitions and assessments

aGVHD was diagnosed and graded based on clinical symptoms and/or skin, oral mucosa, liver or gut biopsy, and disease severity was scored using published consensus criteria.^30^, ^31^, ^32^ Relapse was defined by morphologic evidence of disease in PB, BM, or extramedullary sites or by the recurrence and sustained presence of pre‐transplantation chromosomal abnormalities. Disease‐free survival (DFS) was defined as the probability of being alive and free of disease at any point in time, with death or disease relapse considered events. Overall survival (OS) was defined as the time from transplantation to death from any cause.

### 2.4 Identification and analysis of cell compositions in donor grafts

Samples from G‐BM grafts were labelled with the following monoclonal antibodies and appropriate isotypes: CD45‐PerCP, CD3‐APC, CD4‐PE and CD8‐FITC (BioLegend). The immunophenotype of cell compositions in donor grafts was quantified via flow cytometry. The percentages of CD3^+^ T cells, CD3^+^CD4^+^ T cells and CD3^+^CD8^+^ T cells are expressed as a fraction of low side scatter and CD45^+^ lymphocyte gate. The absolute numbers of graft compositions were calculated as the percentages of these cells multiplied by the percentages of lymphocytes multiplied by the total nucleated cell and divided by the actual patient weight to calculate the numbers of cells per kilogram.

### 2.5 Characterization of monocyte subsets

As previously described,^19^, ^33^, ^34^ classical monocytes, intermediate monocytes and non‐classical monocytes were identified as CD14^high^CD16^−^, CD14^+^CD16^+^ and CD14^+^CD16^high^, respectively. The relative frequencies of these monocyte subsets are expressed as a fraction of the CD14^+^ monocyte subset. Samples from G‐BM grafts were labelled with CD14 and CD16 for monocyte subset analyses. Multiparameter flow cytometric analyses were performed using a BD LSRFortessa cell analyser (BD Biosciences). The data were analysed using BD LSRFortessa software (BD Biosciences). The absolute numbers of monocyte subsets in BM grafts were calculated as the percentages of these cells multiplied by the percentages of total CD14^+^ cells multiplied by the total nucleated cell and divided by the actual patient weight to calculate the numbers of cells per kilogram.

### 2.6 Statistical analysis

Patient variables were compared using the chi‐square test for categorical variables. A Mann‐Whitney U test was performed to analyse continuous variables. Cumulative incidences of aGVHD and relapse were estimated to accommodate competing risks. Death from any cause was defined as a competing risk for aGVHD and relapse. Comparisons between cumulative incidences were performed by the Gray test. The probabilities of OS and DFS were estimated with the Kaplan‐Meier method and compared using the log‐rank test. Multivariate analyses were performed using the Cox proportional hazards model for survival to identify the independent prognostic variables. The parameters with *P* < .1 according to the univariate analysis were entered into a multivariate model. To analyse the association between donor characteristics and graft cell composition, logistic regression analyses were conducted to determine the independent donor factors involved in donor dichotomous variables selected from the univariate analysis. Analyses were performed using GraphPad Prism 6.0 and SPSS (IBM Corporation) version 19 software, and the R software package (version 2.6.1; http://www.r-project.org) was used for competing risk analysis. *P‐*values < .05 were considered statistically significant.

## 3 RESULTS

### 3.1 The percentages and numbers of classical and non‐classical monocytes in BM grafts were different among young, middle‐aged and old donors

**Figure 1:**
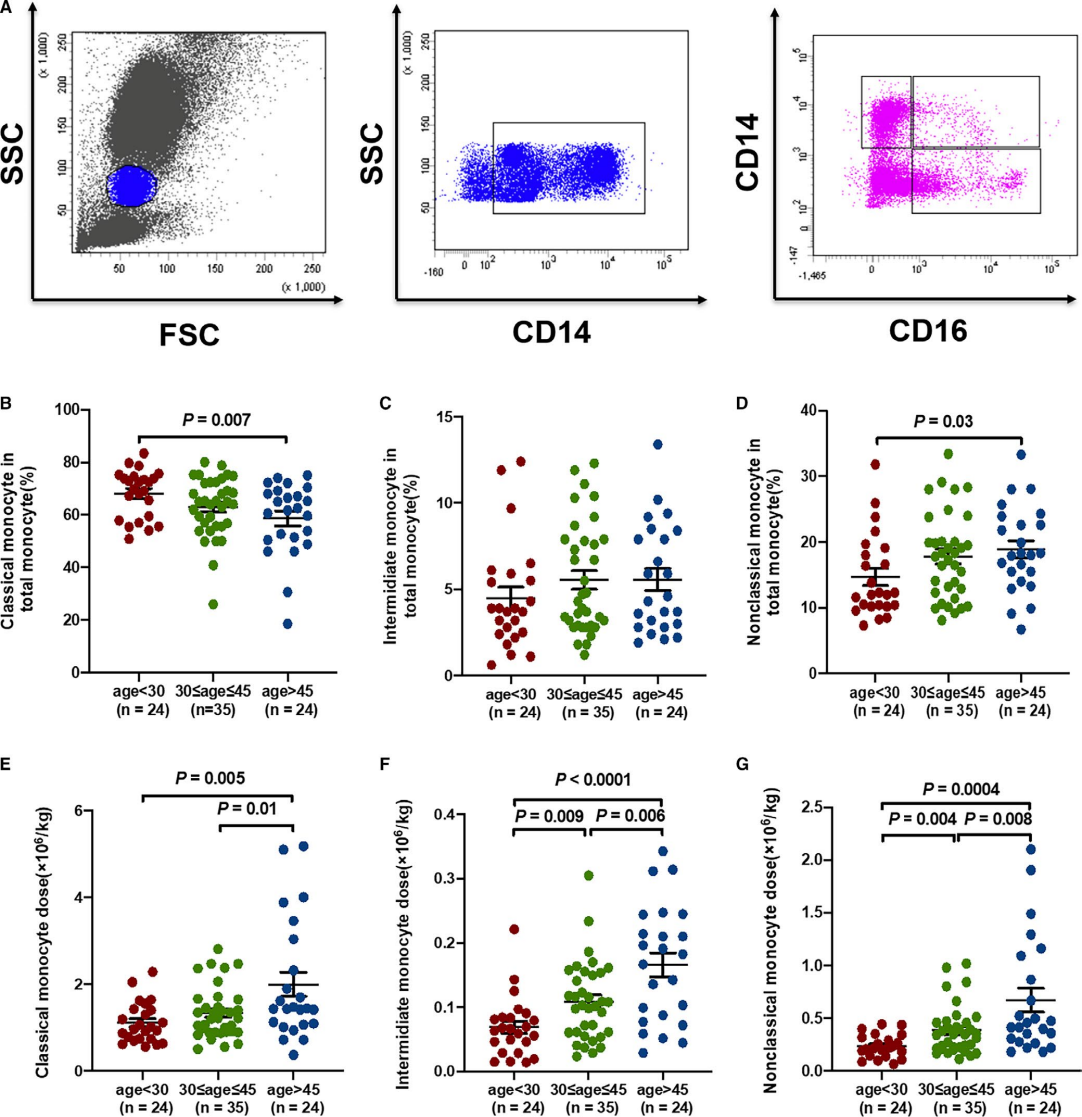
The percentages and numbers of classical and non‐classical monocytes in BM grafts were different in young, middle‐aged and old donors. (A) Representative gating strategy for classical (CD14^high^CD16^−^), intermediate (CD14^+^CD16^+^) and non‐classical monocytes (CD14^+^CD16^high^). Different percentages and numbers of (B, E) classical monocytes, (C, F) intermediate monocytes, and (D, G) non‐classical monocytes among the young (<30 y), middle‐aged (30 y ≤ age≤45 y) and old (>45 y) donor groups. Data are expressed as the mean and standard error of the mean (SEM). All *P*‐values < .05 were considered significant and are provided in the figure

The representative gating strategy for classical, intermediate and non‐classical monocytes in BM grafts is shown in Figure 1A. The enrolled donors were designated into young (age < 30 years), middle‐aged (30 years ≤ age ≤45 years) and old (age > 45 years) groups. Compared with young group, the percentages of classical monocytes in BM grafts (Figure 1B; 58.68%±2.83% vs 68.19%±1.86%; *P* = .007) were significantly lower in old group, whereas the percentages of non‐classical monocytes (Figure 1D; 18.88%±1.32% vs 14.68%±1.28%; *P* = .03) were significantly higher in old group.

Morover, the numbers of classical monocytes (Figure 1E; 2.00 ± 0.28 vs 1.12 ± 0.09; *P* = .005), intermediate monocytes (Figure 1F; 0.17 ± 0.02 vs 0.07 ± 0.01; *P* < .0001) and non‐classical monocytes (Figure 1G; 0.67 ± 0.11 vs 0.23 ± 0.02; *P* = .0004) in BM grafts were significantly higher in old group than those in young group.

### 3.2 Different immune cell subsets in BM grafts among donors of different ages

**Figure 2:**
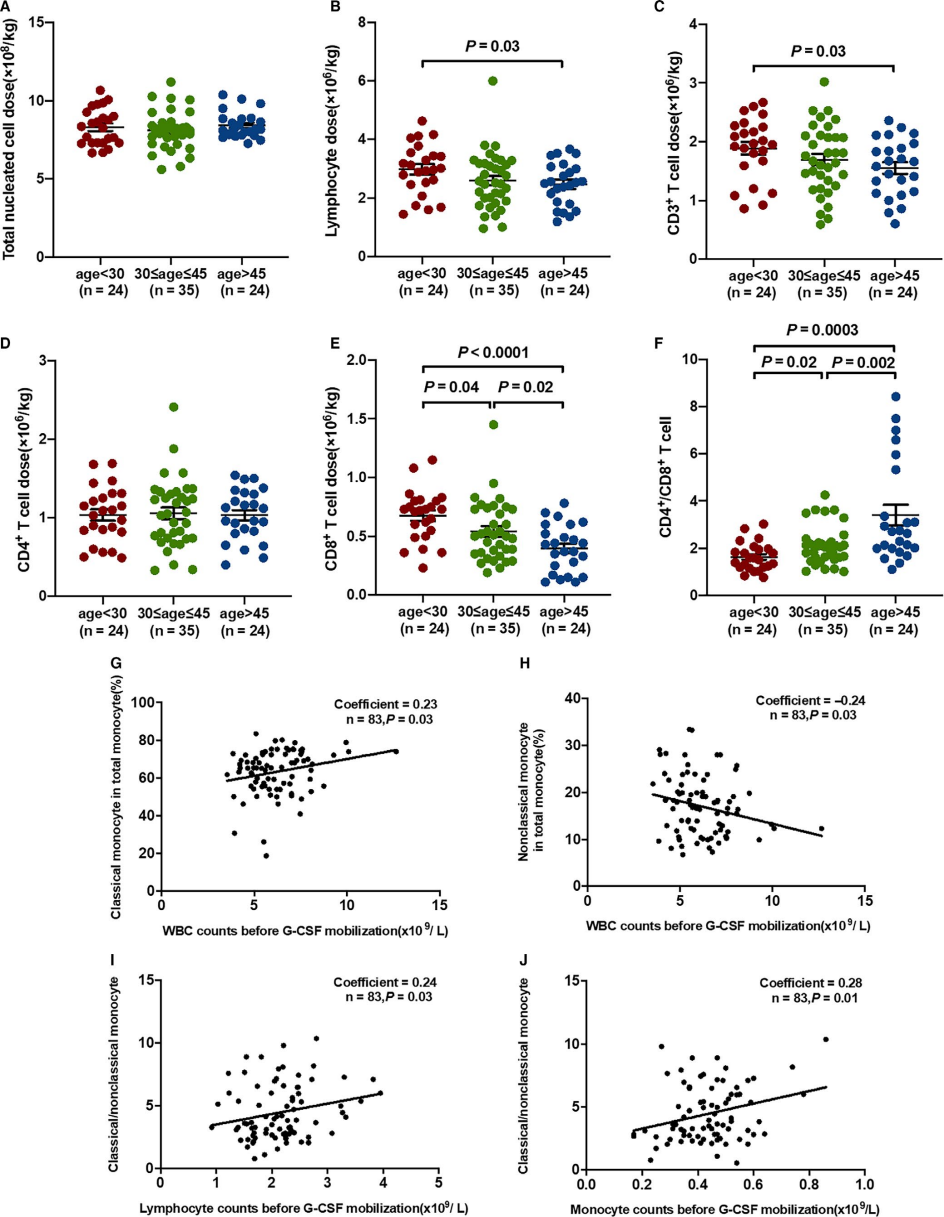
Different immune cell subsets in BM grafts were demonstrated among young, middle‐aged and old donors. (A) Total nucleated cells, (B) lymphocytes, (C) CD3^+^ T cells, (D) CD4^+^ T cells, (E) CD8^+^ T cells, and (F) the ratio of CD4^+^/CD8^+^ T cells were demonstrated among young, middle‐aged and old donor groups. Moreover, WBC counts before G‐CSF mobilization were associated with the percentages of (G) classical monocytes and (H) non‐classical monocytes in BM grafts. (I) Lymphocyte counts and (J) monocyte counts before G‐CSF mobilization were associated with the ratio of classical/non‐classical monocytes in BM grafts. Data are expressed as the mean and standard error of the mean (SEM). All *P‐*values < .05 were considered significant and are provided in the figure

The number of lymphocytes (Figure 2B; 2.47 ± 0.15 vs 2.98 ± 0.18; *P* = .03) and CD3^+^ T cells (Figure 2C; 1.55 ± 0.10 vs 1.89 ± 0.11; *P* = .03) in BM grafts were significantly lower in old group than in young group. Moreover, the number of CD8^+^ T cells in BM grafts was significantly lower in middle‐aged group (Figure 2E; 0.54 ± 0.04 vs 0.68 ± 0.04; *P* = .04) and old group (Figure 2E; 0.40 ± 0.04 vs 0.68 ± 0.04; *P* < .0001) than in young group. Old donors had a lower number of CD8^+^ T cells in BM grafts (Figure 2E; 0.40 ± 0.04 vs 0.54 ± 0.04; *P* = .02) than middle‐aged donors. The numbers of total nucleated cells (Figure 2A) and CD4^+^ T cells (Figure 2D) showed no significant differences among the three age groups. The ratio of CD4^+^/CD8^+^ T cells was significantly higher in middle‐aged group (Figure 2F; 2.11 ± 0.14 vs 1.63 ± 0.12; *P* = .02) and old group (Figure 2F; 3.40 ± 0.44 vs 1.63 ± 0.12; *P* = .0003) than in young group. Therefore, the ratio of CD4^+^/CD8^+^ T cells in BM grafts was highest in old donor group among the three age groups.

### 3.3 WBC counts before G‐CSF mobilization predicted the percentages of classical and non‐classical monocytes in BM grafts

Positive correlations were demonstrated between WBC counts before G‐CSF mobilization and the percentage of classical monocytes (Figure 2G; *r* = .23 (95% confidence interval (CI), 0.02, 0.43); *P* = .03). However, inverse correlations were found between WBC counts before G‐CSF mobilization and the percentage of non‐classical monocytes (Figure 2H; *r* = −.24 (−0.43, −0.02); *P* = .03). In addition, positive correlations were demonstrated between lymphocyte counts before G‐CSF mobilization (Figure 2I; *r* = .24 (0.02, 0.43); *P* = .03), monocyte counts before G‐CSF mobilization (Figure 2J; *r* = .28 (0.07, 0.47); *P* = .01) and the ratio of classical/non‐classical monocytes.

### 3.4 Donor age was independently correlated with monocyte subsets and CD4^+^/CD8^+^ T cells in BM grafts

To clarify the relationship between donor characteristics and monocyte subsets, the ratio of CD4^+^/CD8^+^ T cells in BM grafts, donor age, sex, weight, WBC counts, neutrophils, lymphocytes and monocytes was analysed with univariate and multivariate analyses.

As shown in Table 2, multivariate analysis revealed that donor age ≥ 30 years was associated with high numbers of classical monocyte (2.72, 1.01‐7.35, *P* = .04), intermediate monocyte (9.05, 2.73‐30.00, *P* < .0001) and non‐classical monocytes (7.40, 2.14‐25.57, *P* = .002) in BM grafts. Moreover, donor age ≥ 30 years (6.39, 2.09‐19.54, *P* = .001) was associated a high ratio of CD4^+^/CD8^+^ T cells in BM grafts.

**Table 2:** Univariate and multivariate analysis of donor characteristic effect on the monocyte subsets and CD4^+^/CD8^+^ T cells in BM grafts

Factors	Univariate analysis	Multivariate analysis^a^		
	*P‐*value	HR	95% CI	*P‐*value
Transplanted classical monocyte dose (×10^6^/kg)				
Donor age, <30 vs ≥30	0.03	2.72	1.01‐7.35	.04
Transplanted intermediate monocyte dose (×10^6^/kg)				
Donor age, <30 vs ≥30	0.0005	9.05	2.73‐30.00	<.0001
Transplanted non‐classical monocyte dose (×10^6^/kg)				
Donor age, <30 vs ≥30	0.002	7.40	2.14‐25.57	.002
CD4^+^/CD8^+^ T cells				
Donor age, <30 vs ≥30	0.004	6.39	2.09‐19.54	.001

Abbreviations: G‐CSF, granulocyte colony‐stimulating factor; WBC, white blood cell.

^a^ To avoid potential confounding factors, logistic regression was assessed for interaction terms with covariates. The variables included in the logistic regression analyses exhibited *P* < .10 after univariate analyses. The final multivariate models were constructed using a forward stepwise selection approach.

### 3.5 Percentages and numbers of classical, intermediate and non‐classical monocytes in BM grafts of grade II‐IV aGVHD patients

As shown in Table 3, most of the demographic and clinical characteristics showed no significant differences between patients with grade 0‐I aGVHD and those with grade II‐IV aGVHD.

**Table 3:** Characteristics of allo‐HSCT patients with grade 0‐I aGVHD and grade II‐IV aGVHD

Characteristics	Grade 0‐I aGVHD (n = 57)	Grade II‐IV aGVHD (n = 26)	*P‐*value*
Age at HSCT (y)	34 (18‐55)	36 (19‐59)	.66
Gender (male/female)	35/22	15/11	1
Underlying disease			.46
AML	39	16	
ALL	16	8	
MDS	2	2	
Status at HSCT			1
Standard‐risk	46	21	
High‐risk	11	5	
Source of stem cell			1.00
BM and PB	57	26	
Transplanted total nucleated cell dose (×10^8^/kg)^a^	8.17 (6.48‐11.19)	7.99 (5.60‐10.66)	.29
Transplanted lymphocyte dose (×10^6^/kg)^a^	2.62 (0.96‐6.00)	2.69 (1.01‐4.12)	.90
Transplanted CD3^+^ cell dose (×10^6^/kg)^a^	1.67 (0.59‐3.32)	1.94 (0.69‐2.53)	.30
Transplanted CD4^+^ cell dose (×10^6^/kg)^a^	0.97 (0.33‐2.41)	1.16 (0.34‐1.68)	.24
Transplanted CD8^+^ cell dose (×10^6^/kg)^a^	0.50 (0.11‐1.45)	0.58 (0.11‐1.15)	.55
Transplanted CD14^+^ cell dose (×10^6^/kg)^a^	1.55 (0.57‐3.01)	1.44 (0.69‐2.68)	.68
Donor match			1.00
HLA‐partially matched related donor	57	26	
ABO mismatch			
No	32	15	.78
Minor	24	11	1.00
Major	1	0	.31
Pre‐HSCT cycles of chemotherapy	4 (1‐10)	4 (1‐10)	.36
Conditioning			1.00
BU/CY + ATG	57	26	
History of CMV reactivation	44	21	.78
History of refractory CMV reactivation	22	13	.35

Abbreviations: aGVHD, acute graft‐vs‐host disease; ALL, acute lymphocytic leukaemia; allo‐HSCT, allogeneic haematopoietic stem cell transplantation; AML, acute myelogenous leukaemia; BM, bone marrow; CMV, cytomegalovirus; HLA, human leukocyte antigen; MDS, myelodysplastic syndrome; PB, peripheral blood.

^a^ Data are reported as median (range).

* The continuous variables were compared using the Mann‐Whitney *U* test, and the differences between the two groups were compared using the chi‐square test. The criterion for statistical significance was *P* < .05.

**Figure 3:**
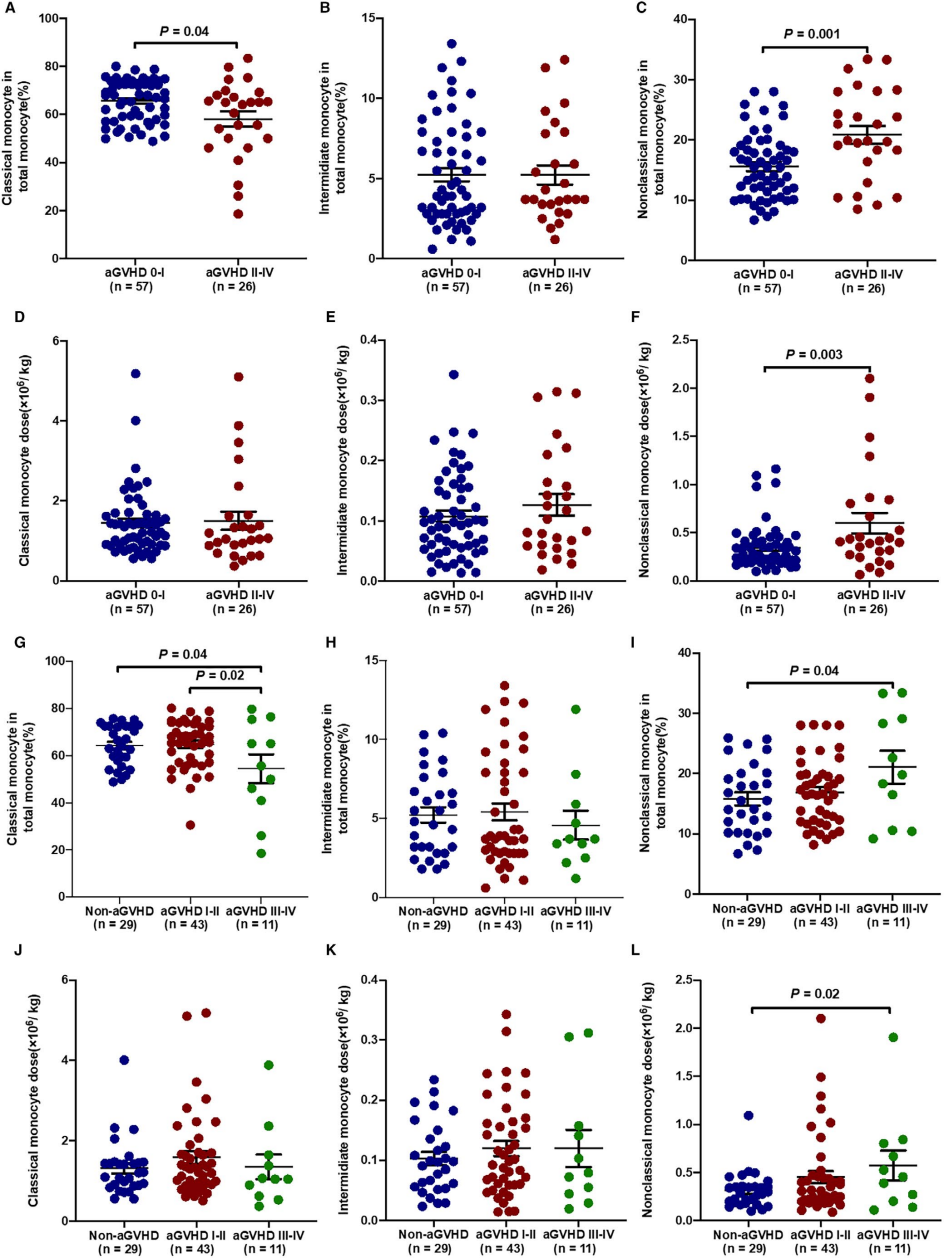
Monocyte subsets in BM grafts of aGVHD patients. Different percentages and numbers of (A, D) classical monocytes, (B, E) intermediate monocytes, and (C, F) non‐classical monocytes in BM grafts between grade 0‐I and grade II‐IV aGVHD patients. Moreover, different percentages and numbers of (G, J) classical monocytes, (H, K) intermediate monocytes and (I, L) non‐classical monocytes in BM grafts between grade I‐II and grade III‐IV aGVHD patients. Data are expressed as the mean and standard error of the mean (SEM). All *P‐*values < .05 were considered significant and are provided in the figure

As illustrated in Figure 3, when compared with grade 0‐I aGVHD patients, the percentage of classical monocytes (Figure 3A; 58.15%±3.16% vs 65.61%±1.16%; *P* = .04) was significantly lower in grade II‐IV aGVHD patients, whereas the percentage of non‐classical monocytes (Figure 3C; 20.85%±1.47% vs 15.54%±0.72%; *P* = .001) was significantly higher in grade II‐IV aGVHD patients. Moreover, the number of non‐classical monocytes (Figure 3F; 0.60 ± 0.11 vs 0.34 ± 0.03; *P* = .003) was significantly higher in grade II‐IV aGVHD patients than those with grade 0‐I aGVHD patients.

### 3.6 Percentages and numbers of classical, intermediate and non‐classical monocytes in BM grafts affect the severity of aGVHD

To evaluate whether the severity of aGVHD is associated with the level of monocytes in BM grafts, the percentages and numbers of classical monocytes, intermediate monocytes and non‐classical monocytes were compared between patients with grade III‐IV aGVHD and those with grade I‐II aGVHD. The percentage of classical monocytes was significantly lower in grade III‐IV aGVHD patients than in grade I‐II aGVHD patients (Figure 3G; 54.53%±6.18% vs 64.73%±1.56%; *P* = .02) and non‐aGVHD patients (Figure 3G; 54.53%±6.18% vs 64.21%±1.61%; *P* = .04), whereas the percentage of non‐classical monocytes (Figure 3I; 21.05%±2.72% vs 15.80%±1.08%; *P* = .04) was significantly higher in grade III‐IV aGVHD patients than those with non‐aGVHD patients. Moreover, the number of non‐classical monocytes (Figure 3L; 0.57 ± 0.15 vs 0.31 ± 0.04; *P* = .02) was significantly higher in grade III‐IV aGVHD patients than those with non‐aGVHD patients.

### 3.7 The monocyte subsets in BM grafts were associated with the incidence of grade II‐IV aGVHD but did not have a significant influence on relapse or survival

The enrolled patients were designated into the high BM graft group or the low BM graft group according to the median numbers of the transplanted classical monocytes (1.22 × 10^6^/kg), intermediate monocytes (0.10 × 10^6^/kg) or non‐classical monocytes (0.31 × 10^6^/kg) in BM grafts.

**Figure 4:**
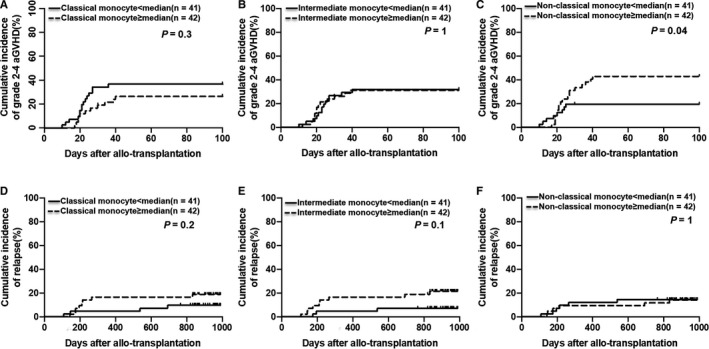
Effects of classical, intermediate and non‐classical monocytes in BM grafts on grade II‐IV aGVHD and relapse. The ‘low’ and ‘high’ groups were separated according to the median numbers of classical, intermediate and non‐classical monocytes in BM grafts. Effects of (A) classical monocytes, (B) intermediate monocytes and (C) non‐classical monocytes in BM grafts on grade II‐IV aGVHD. Effects of (D) classical monocytes, (E) intermediate monocytes and (F) non‐classical monocytes in BM grafts on relapse. The cumulative incidences of grade II‐IV aGVHD and relapse were calculated. Competing risks were accounted for using Gray's test

The cumulative incidence of grade II‐IV aGVHD in low non‐classical monocyte group was significantly lower than that in high non‐classical monocyte group (Figure 4C; 19.5% (9.4%‐35.4%) vs 42.9% (28.1%‐58.9%), *P* = .04). After a median follow‐up of 764 days (range 49‐989 days), the cumulative incidence of relapse (Figure 4D‐F) and the probabilities of DFS and OS (Figure S1) showed no significant differences between the different monocyte subsets groups.

### 3.8 Non‐classical monocytes in BM grafts were an independent risk factor for the occurrence of grade II‐IV aGVHD

As shown in Table 4, the association between donor characteristics and the occurrence of grade II‐IV aGVHD was analysed with a univariate analysis. The percentage of classical monocytes in BM grafts was negatively correlated with the incidence of grade II‐IV aGVHD. However, the percentage of non‐classical monocytes in BM grafts was positively correlated with the incidence of grade II‐IV aGVHD. Multivariate analysis demonstrated that non‐classical monocytes in BM grafts, which accounted for ≥0.31 × 10^6^/kg (2.32, 1.01‐5.33, *P* = .04), was independently correlated with a high incidence of grade II‐IV aGVHD after allo‐HSCT.

**Table 4:** Univariate and multivariate analyses of risk factors for the occurrence of grade II‐IV aGVHD after allo‐HSCT

Factors	Univariate analysis	Multivariate analysis^a^		
	*P‐*value	HR	95% CI	*P‐*value
Donor gender, female vs male	.30			
Donor age, <30 vs ≥30	.80			
Donor weight (kg) <69 vs ≥69	.48			
Donor BMI (kg/m^2^) <23.8 vs ≥23.8	1			
Transplanted total nucleated cell dose (×10^8^/kg) <8.15 vs ≥8.15	.35			
Transplanted lymphocyte dose (×10^6^/ kg) <2.62 vs ≥2.62	1			
Transplanted CD3^+^ cell dose (×10^6^/kg) <1.80 vs ≥1.80	.24			
Transplanted CD4^+^ cell dose (×10^6^/kg) <1.05 vs ≥1.05	.24			
Transplanted CD8^+^ cell dose (×10^6^/kg) <0.51 vs ≥0.51	.81			
Transplanted CD4^+^/CD8^+^ cell ratio < 1.99 vs ≥1.99	.81			
Transplanted CD14^+^ cell dose (×10^6^/kg) <1.52 vs ≥1.52	.16			
Transplanted classical monocyte dose (×10^6^/kg) <1.22 vs ≥1.22	.28			
Transplanted intermediate monocyte dose (×10^6^/kg) <0.10 vs ≥0.10	.99			
Transplanted non‐classical monocyte dose (×10^nn^/kg) <0.31 vs ≥0.31	.04	2.32	1.01‐5.33	.04

Abbreviations: aGVHD, acute graft‐vs‐host disease; allo‐HSCT, allogeneic haematopoietic stem cell transplantation;CI, confidence interval; HR, hazard ratio.

^a^ To avoid potential confounding factors, multivariate Cox proportional hazard models were assessed for interaction terms with covariates. The variables included in the Cox models exhibited *P* < .10 after univariate analyses. The final multivariate models were constructed using a forward stepwise selection approach.

## 4 DISCUSSION

In the current study, we found that classical monocytes were significantly increased in donors <30 years old, whereas non‐classical monocytes and the ratio of CD4^+^/CD8^+^ T cells were remarkably decreased in BM grafts in donors <30 years old. In addition, patients who received a BM graft with a high proportion of non‐classical monocytes exhibited a significantly high incidence of aGVHD, whereas the percentage of classical monocytes in BM grafts was negatively correlated with the incidence of aGVHD. Multivariate analysis further demonstrated that non‐classical monocytes in BM grafts (≥0.31 × 10^6^/kg) were independently correlated with a high incidence of grade II‐IV aGVHD after allo‐HSCT.

Previous work revealed that young donors are correlated with a low incidence of aGVHD.^4^, ^5^, ^6^, ^8^, ^9^, ^10^ However, the underlying reason why young donors are associated with a lower incidence of aGVHD than old donors remains to be clarified. Several cell compositions in grafts have been reported to be useful for identifying patients at high risk for aGVHD. Luo et al found that a CD4^+^/CD8^+^ T cell ratio ≥1.16 in BM grafts was associated with a high risk for aGVHD.^11^ Subsequently, in a controlled, open‐label, randomized trial, prophylaxis with a low‐dose corticosteroid for high‐risk patients who were infused with BM grafts at a CD4^+^/CD8^+^ T cell ratio of ≥1.16 significantly decreased the incidence and delayed the onset of aGVHD.^35^ Moreover, our previous study found that young donors were associated with a higher number of CD14^+^ monocytes in donor grafts.^17^ The current study provides new evidence that BM grafts harvested from young donors contain low percentages of non‐classical monocytes but high percentages of classical monocytes. Moreover, BM grafts harvested from young donors contain low number of non‐classical monocytes. Yakoub‐Agha et al reported that the ratio of CD4^+^/CD8^+^ T cells in grafts is positively associated with donor age.^16^ We confirmed that young donors contain a lower ratio of CD4^+^/CD8^+^ T cells in grafts. Although further validation is required, our data indicate that the different cell compositions in BM grafts, which are characterized by the low level of non‐classical monocytes and a low ratio of CD4^+^/CD8^+^ T cell in BM grafts, may be correlated with the lower incidence of aGVHD in young donors.

Circulating monocytes play an important role in innate and adaptive immune resaponses and are involved in the pathogenesis of inflammatory disorders. Further research has successfully characterized non‐classical monocytes as proinflammatory cells with high endothelial affinity. Under LPS stimulation, classical monocytes secrete a large amount of G‐CSF, IL‐10, IL‐6 and CCL2, whereas non‐classical monocytes secrete a large amount of TNF‐α and IL‐1β.^24^, ^25^ TNF‐α participates in the initiating events that culminate in aGVHD and amplify the disease process once established.^36^, ^37^ In vitro research has suggested that CD16^+^ human or mouse monocytes can drive the expansion of T cells in a mixed lymphocyte reaction better than CD16^−^ monocytes.^38^ Moreover, previous studies have suggested an imbalance in monocyte differentiation in aGVHD patients.^21^, ^23^ Our multivariate analysis further revealed that the level of non‐classical monocytes in BM grafts (≥0.31 × 10^6^/kg) was an independent risk factor for the occurrence of II‐IV aGVHD after allo‐HSCT. Based on previous reports and the current study, we speculate that non‐classical monocytes may play an important role in the occurrence of aGVHD by inducing TNF‐α production and promoting the induction of proinflammatory cells. Therefore, further functional studies are needed to elucidate the underlying mechanism of non‐classical monocytes in BM grafts affecting the occurrence of aGVHD.

However, we are aware that further studies are needed to clarify the effect of monocyte subsets in PB grafts on aGVHD. Moreover, further clarification is required to determine whether an imbalance in monocytes in grafts directly affects or participates in immunoregulatory effects on other immune cells in aGVHD after allo‐HSCT.

In summary, the current study shows that donor age is positively correlated with the percentage of non‐classical monocytes and the ratio of CD4^+^/CD8^+^ T cells, whereas negatively correlated with the percentage of classical monocytes in BM grafts. Moreover, our data indicate that non‐classical monocytes in BM grafts may help to identify patients who are at high risk for aGVHD after allo‐HSCT. Although further validation is required, our results suggest that the low level of non‐classical monocytes and a low ratio of CD4^+^/CD8^+^ T cell in BM grafts may be correlated with the lower incidence of aGVHD in young donors.

## CONFLICT OF INTEREST

The authors declare that they have no competing interests.

## AUTHOR CONTRIBUTIONS

**Qi Wen:** Formal analysis (equal); Investigation (lead); Methodology (lead); Project administration (lead); Validation (equal); Writing‐original draft (lead); Writing‐review & editing (lead). **Hongyan Zhao:** Formal analysis (equal); Investigation (equal); Project administration (equal); Validation (equal); Writing‐original draft (equal); Writing‐review & editing (equal). **Wei‐Li Yao:** Formal analysis (equal); Investigation (equal); Methodology (equal); Project administration (equal); Validation (equal); Writing‐original draft (equal); Writing‐review & editing (supporting). **Yuanyuan Zhang:** Formal analysis (equal); Investigation (equal); Project administration (equal); Validation (equal). **Hai‐Xia Fu:** Formal analysis (equal); Investigation (equal); Project administration (equal); Supervision (supporting). **Yu Wang:** Formal analysis (supporting); Investigation (supporting); Project administration (supporting); Supervision (supporting). **Lan‐Ping Xu:** Formal analysis (supporting); Investigation (supporting); Project administration (supporting); Supervision (supporting). **Xiaohui Zhang:** Formal analysis (supporting); Investigation (supporting); Project administration (supporting); Supervision (supporting). **Yuan Kong:** Conceptualization (lead); Data curation (equal); Formal analysis (lead); Funding acquisition (lead); Investigation (lead); Methodology (equal); Project administration (lead); Supervision (lead); Validation (lead); Writing‐original draft (lead); Writing‐review & editing (lead). **Xiao‐Jun Huang:** Conceptualization (lead); Data curation (equal); Formal analysis (lead); Funding acquisition (lead); Investigation (lead); Project administration (lead); Supervision (lead); Validation (lead); Writing‐original draft (equal); Writing‐review & editing (lead).

## Supporting information

Fig S1

## Data Availability

The data that support the findings of this study are available from the corresponding author upon reasonable request.
