# Supplementary material for: Monocyte subsets in bone marrow grafts may contribute to a low incidence of acute graft‐vs‐host disease for young donors
Source: J Cell Mol Med. 2020 Jun 30;24(16):9204–16. doi: 10.1111/jcmm.15557 (PMC7417711; doi:10.1111/jcmm.15557)
Supplement: Supplementary file 1 — Fig S1 [file JCMM-24-9204-s001.docx]

**Supplementary Materials**

**Monocyte Subsets in Bone Marrow Grafts May Contribute to a Low Incidence of Acute Graft-versus-Host Disease for Young Donors**

**Authors:** Qi Wen^1,2^, Hong-Yan Zhao^2^, Wei-Li Yao^2^, Yuan-Yuan Zhang^2^, Hai-Xia Fu^2^, Yu Wang^2^, Lan-Ping Xu^2^, Xiao-Hui Zhang^2^, Yuan Kong^2^*, Xiao-Jun Huang^1,2^*

*Xiao-Jun Huang and Yuan Kong are co-correspondence authors.

**
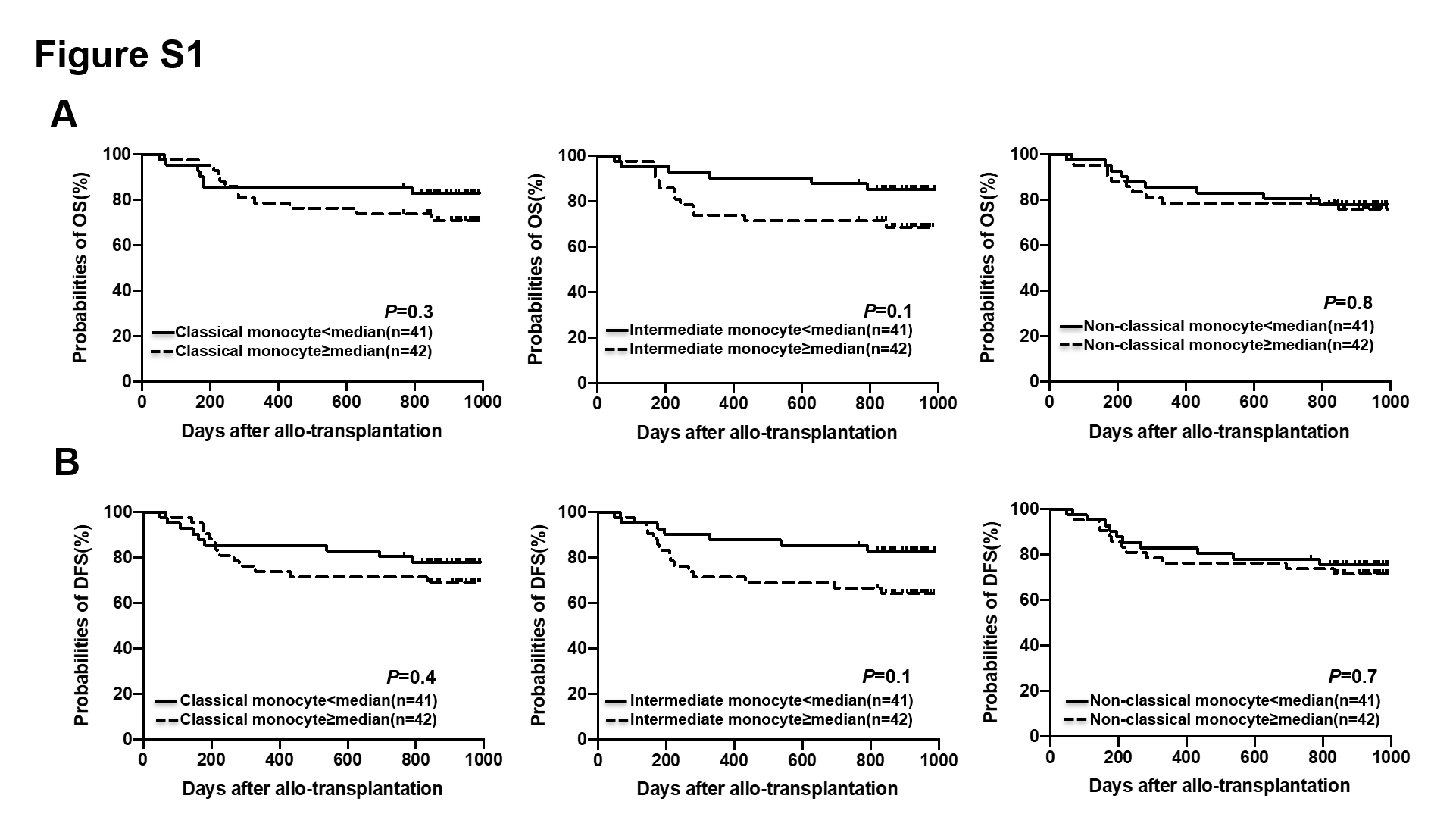
**

**Figure S1: Effect of classical, intermediate and non-classical monocyte in BM grafts on DFS and OS.** The “low” and “high” groups were separated according to the median classical, intermediate and non-classical monocyte in BM grafts. **A**. OS; **B**. DFS. OS and DFS probabilities were estimated using the Kaplan-Meier method and were compared using the log-rank test.
